# Genetic Proxies for *Helicobacter pylori* Serology Are Dominated by HLA Class II Haplotype Structure: Implications for Reported Associations with Neurodegenerative Disease

**DOI:** 10.3390/microorganisms14102193

**Published:** 2026-10-01

**Authors:** Hyuk Lee, Yang Won Min, Tae Jun Kim

**Affiliations:** 1 Department of Medicine, Samsung Medical Center, Sungkyunkwan University School of Medicine, Seoul 06351, Republic of Korea; 2 Samsung Precision Genome Medicine Institute, Samsung Medical Center, Seoul 06351, Republic of Korea; 3 Division of Gastroenterology, Department of Internal Medicine, Hallym University Dongtan Sacred Heart Hospital, Hallym University College of Medicine, Hwaseong 18450, Republic of Korea

**Keywords:** *Helicobacter pylori*, serology, HLA class II, major histocompatibility complex, Mendelian randomisation, linkage disequilibrium, instrumental variables, neurodegenerative disease

## Abstract

Genetic variants that predict anti-*Helicobacter pylori* immunoglobulin G (IgG) seropositivity lie almost entirely within the major histocompatibility complex (MHC), which also carries established risk loci for several diseases. Mendelian randomisation studies using such variants have reported causal effects of *H. pylori* infection on neurodegenerative disease, although the phenotype indexed is anti-*H. pylori* IgG seropositivity, not infection itself. We evaluated 22 candidate variants from a Japanese genome-wide study of anti-*H. pylori* IgG antibody titre in two-sample Mendelian randomisation analyses across five prespecified genomic proxy sets; the number retained varied by outcome. Higher genetically predicted anti-*H. pylori* IgG seropositivity was associated with lower Alzheimer’s disease risk (odds ratio 0.83, 95% confidence interval 0.73 to 0.95). The association was absent once MHC variants were removed (0.94, 0.78 to 1.12), was strongest using HLA class II variants alone (0.73, 0.59 to 0.91), and traced to two variants near *HLA-DRB1* and *HLA-DQA1*. Those proxies did not predict peptic ulcer disease in an ancestry-matched Japanese study (1.02, 0.92 to 1.13), differing from Parkinson’s disease (0.73, 0.55 to 0.97; difference *p* = 0.029). Colocalisation did not support a shared causal variant for the serological and disease signals, selected proxy associations were attenuated after conditioning on leading class II variants, and the haplotypes tagged by the proxies differed between European and East Asian reference panels, indicating limited cross-ancestry transferability. The behaviour of these proxies is dominated by HLA class II haplotype structure, and they are therefore unsuitable for inferring a protective effect of *H. pylori* infection on neurodegenerative disease from IgG seropositivity.

## 1. Introduction

*Helicobacter pylori* colonises the stomach of roughly half the world’s population [1] and is an established cause of peptic ulcer disease and gastric cancer [2]. Attention has also turned to conditions outside the gut: recent meta-analyses have reported that *H. pylori* infection is more common in Parkinson’s disease, dementia and Alzheimer’s disease [3,4]. Clinical interest has been sustained by evidence that eradication lengthens levodopa on-time in patients with motor fluctuations [5], although a later placebo-controlled trial found no improvement in motor, non-motor or quality-of-life outcomes [6].

These findings are difficult to interpret. Gastrointestinal dysfunction is common in Parkinson’s disease, and patients undergoing investigation may be more likely to receive endoscopy or testing, creating opportunities for reverse causation and differential ascertainment. Studies using inherited genetic variation can reduce these problems, but only when the variants remain valid proxies for the exposure and satisfy the instrumental-variable assumptions. Since genome-wide data on antibody responses became available, several such studies have appeared. Their conclusions do not agree: two studies drawing on the same panel of 46 antibody traits reported different positive findings, one for Alzheimer’s disease [7] and one for Parkinson’s disease [8]. Of twelve such studies identified by the systematic PubMed search described in Section 2.8 and read in full [7,8,9,10,11,12,13,14,15,16,17,18], none mentioned the major histocompatibility complex (MHC) or chromosome 6 anywhere in the text, and none excluded or partitioned the region, whereas a similar study using protein rather than antibody measurements discussed the region five times and excluded it from its primary analysis [19].

Anti-*H. pylori* IgG serology is not a direct measure of active infection because antibodies may persist after bacterial clearance or eradication [2]. Serology also reflects how efficiently a person presents the relevant antigens, a process governed largely by the human leukocyte antigen (HLA) genes; the original antibody study noted that 14 of its 46 traits had their strongest signal in the MHC [20]. If the assay phenotype is dichotomised, a Mendelian randomisation estimate pertains to liability underlying seropositivity rather than directly to the effect of changing infection status [21]. The outcomes are governed by the same region, Parkinson’s disease is associated with the HLA-DRB1*04 haplotype [22,23], Alzheimer’s disease with an adjacent interval between *HLA-DRB1* and *HLA-DQA1* [23], and amyotrophic lateral sclerosis with a class II variant of its own [24], the three signals lying within about 100 kilobases. In multiple sclerosis, the MHC contains the dominant common genetic association [25], making it a useful reference point: variants indexing a class II haplotype should be strongly associated with the disease, whereas variants indexing exposure to *H. pylori* need not be.

When exposure and outcome are governed by the same narrow stretch of the genome, the assumption that a proxy affects the outcome only through the exposure becomes questionable. Two explanations are then available for the reported association: a causal effect of *H. pylori* infection, or linkage disequilibrium (the non-random co-inheritance of neighbouring alleles) between the antibody proxies and HLA class II risk haplotypes, a recognised concern in Mendelian randomisation studies of infection [26]. We set out to distinguish these explanations and to identify the features of the analysis that determine the answer. Which explanation holds bears on whether eradication warrants evaluation as a preventive strategy and on how serological proxies should be used elsewhere.

## 2. Materials and Methods

### 2.1. Genetic Proxies for Anti-H. pylori IgG Seropositivity

We used variants identified in a genome-wide study of anti-*H. pylori* IgG antibody titre in six Japanese cohorts contributing fourteen datasets [27] (125,178 participants). In that study, titre was measured by enzyme-linked immunosorbent assay (E-Plate Eiken *H. pylori* antibody, Eiken Chemical, Tokyo, Japan) or by latex agglutination turbidimetry (*H. pylori*-latex Seiken, Denka Seiken, Tokyo, Japan) and, because the distribution of titre is strongly skewed, it was dichotomised at a cohort-specific cut-off, either the top quartile of the titre distribution or 3 U/mL. The combined meta-analysis from which our instruments are taken compared 37,410 participants above that cut-off with 87,768 below it, a case fraction of 29.9%. A unified 3 U/mL threshold applied to every cohort (58,106 against 67,072) was one of that study’s sensitivity analyses and is not the analysis used here; we note that the source reports the latter figures once in its Results Section as though they described the main analysis, whereas its Methods Section, its Table 1 and its figure legends give 37,410 and 87,768. Association testing was adjusted for age, sex and the first ten principal components, using a linear mixed model in the cohorts containing related individuals and fixed-effect inverse-variance weighted meta-analysis across cohorts. In the Tohoku Medical Megabank cohorts, genotypes were imputed against a combined 3.5KJPNv2 and 1000 Genomes phase 3 reference panel and filtered at an imputation score of 0.4 and a minor allele frequency of 0.01; the remaining cohorts were genotyped, imputed and analysed separately under the study’s common protocol. The phenotype was validated in that study against the pepsinogen I-to-II ratio and against self-reported infection history. We use seropositivity throughout as shorthand for a titre above this cut-off. The exposure is therefore liability to a high antibody response, which stands one step further from infection status than a clinically defined seropositive result would. As detailed in the Supplementary Methods, the reported effects were on the log-odds scale, so we interpret the exposure as liability to IgG seropositivity rather than as a standard-deviation change in continuous antibody titre.

Twenty-two candidate variants were initially selected. The reported all-variant estimates included 13–16 variants across neurodegenerative outcome datasets and 21 variants for each peptic ulcer outcome; outcome-specific counts are provided in Supplementary Materials Table S7. All 22 reached *p* < 5 × 10^−8^, were reported as independent after clumping at *r*^2^ below 0.05, and were strong by standard criteria (*F* 28 to 146); each is listed in Supplementary Materials Table S1. Full summary statistics have not been released, so we used the reported values for the lead variants. Effect alleles were aligned between exposure and outcome before analysis; palindromic variants were resolved by allele frequency where this was unambiguous and dropped otherwise, and two positions misreported in the source publication were corrected against dbSNP. Harmonisation and exclusions are in the Supplementary Methods, Supplementary Materials Table S7 and, variant by variant, Supplementary Materials Table S20.

Where a proxy was absent from an outcome dataset we did not substitute a correlated variant. A substitute is chosen on its correlation with the missing variant in a reference panel, and in this region that correlation extends over long distances and differs between populations, so a substitute selected in a European panel may tag a different class II haplotype from the variant it replaces. Since what these variants tag is the question at issue, admitting substitutes would introduce the very error the analysis sets out to measure. Two outcome datasets were retrieved through a programmatic interface whose default behaviour is to fill missing variants with linkage-disequilibrium proxies; on audit we found that this default had been left in place for Parkinson’s disease and East Asian Parkinson’s disease, substituting five variants and one variant, respectively, with four of the five within HLA class II. We have disabled it and report those two analyses without substitution. The corresponding estimates using substitutes are given in Supplementary Materials Table S19 and differ little, but they are not the analysis we describe here. The number of variants contributing to each estimate is reported with every result. A row-by-row record of how every candidate variant was matched and aligned in each outcome dataset is given in Supplementary Materials Table S20. For the six neurodegenerative outcomes, that table also states whether each variant entered the reported analysis. For the three peptic ulcer outcomes a per-variant record of the reported analysis was not exported, so the table gives the harmonisation audit only and the counts in Supplementary Materials Table S7 remain the reference for those outcomes.

### 2.2. Outcome Datasets

All outcome data were summary results from published genome-wide association studies of Parkinson’s disease, Alzheimer’s disease, amyotrophic lateral sclerosis, East Asian Parkinson’s disease, peptic ulcer disease and multiple sclerosis (Table 1) [24,28,29,30,31,32]. The multiple sclerosis statistics were obtained from the International Multiple Sclerosis Genetics Consortium under its data-access procedure and were used only for individual-variant estimates and the density of signal across the region [30]. No outcome dataset adjusted for a trait plausibly on the pathway from the serological phenotype to disease, so collider bias through such adjustment does not apply.

**Table 1:** Characteristics of the outcome datasets.

Outcome	Source	Accession	Ancestry	Cases	Controls	Genome Build
Parkinson’s disease (primary)	Nalls et al., 2019 [31]	ieu-b-7	European	33,674	449,056	GRCh37
Parkinson’s disease (no proxy cases)	Nalls et al., 2019 [31]	GCST009324	European	15,056	12,637	GRCh37
Alzheimer’s disease (primary)	Consensus meta-analysis, 2026 [28]	GCST90704647	European	75,638	1,043,805	GRCh38
Alzheimer’s disease (clinically ascertained)	Consensus meta-analysis, 2026 [28]	GCST90704648	European	56,184	90,919	GRCh38
Amyotrophic lateral sclerosis	van Rheenen et al., 2021 [24]	GCST90027164	European	27,205	110,881	GRCh37
Parkinson’s disease (East Asian)	Biobank Japan [32]	GCST90018674	East Asian	340	175,788	GRCh37
Peptic ulcer disease (positive control)	He et al., 2023 [29]	GCST90270926	Japanese	29,739	240,675	GRCh37
Gastric ulcer	He et al., 2023 [29]	GCST90270927	Japanese	20,972	240,675	GRCh37
Duodenal ulcer	He et al., 2023 [29]	GCST90270928	Japanese	11,964	240,675	GRCh37
Multiple sclerosis (HLA class II comparison)	IMSGC discovery meta-analysis [30]	By application	European	14,802	26,703	GRCh37

The two Parkinson’s disease datasets differ according to whether cases identified through an affected first-degree relative were included; the second excludes those proxy cases and also excludes 23andMe participants, and is a separate release (GCST009324) rather than a subset of the primary file. The Alzheimer’s disease datasets differ according to whether biobank cohorts were included; the clinically ascertained analysis was restricted to diagnosed cases. Case and control counts are those reported by the original data sources. IMSGC denotes the International Multiple Sclerosis Genetics Consortium.

### 2.3. Sets of Proxies Defined by Genomic Location

We repeated every analysis using five sets of proxies all on GRCh37, defined in advance: all of them; those outside the extended MHC (chromosome 6, 25 to 34 megabases); those inside it; those within class I (29.6 to 31.5 megabases); and those within class II (32.3 to 33.1 megabases). The same sets were applied to every exposure and outcome, so none was chosen after seeing a result. An alternative published definition of the boundaries was used as a sensitivity analysis.

### 2.4. Estimation

Mendelian randomisation requires that the proxies predict the exposure, share no cause with the outcome, and affect the outcome only through the exposure. We assessed the first with per-variant *F* statistics and their arithmetic means within each outcome-specific proxy set and the second by looking up every proxy in the GWAS Catalog; the third cannot be verified directly and is the subject of the analyses that follow.

Our main estimate was the multiplicative random-effects inverse-variance weighted average of the individual-variant ratios, reported alongside the weighted median, MR-Egger, weighted mode, MR-cML, MR-Lasso and contamination mixture estimators [33,34,35,36,37,38]. Heterogeneity was assessed with Cochran Q, directional pleiotropy with the MR-Egger intercept and MR-PRESSO, and the assumed direction of effect with the Steiger test [39,40]. Because the proxies do not remain independent in Europeans, selected inverse-variance weighted analyses were repeated using a reference correlation matrix matched to the ancestry of each outcome (European or East Asian), restricted to variants available in the corresponding panel (Supplementary Materials Table S2). Results are odds ratios with 95% confidence intervals, *p* values and false discovery rate *q* values. Because the five proxy sets are nested by design and were specified in advance, the primary *H. pylori* analyses used Benjamini–Hochberg correction across the six outcomes within each proxy set. The additional pathogen-antibody traits formed a separate family: within each proxy set, Benjamini–Hochberg correction was applied across every antibody-trait and outcome combination for which an estimate could be obtained, giving 29, 21, 16, 12 and 8 tests for the all-variant, MHC-excluded, MHC-only, class I-only and class II-only sets (Supplementary Materials Table S5). Combinations with no usable proxy were excluded from the family rather than counted as tests. The peptic ulcer analyses are reported with nominal *p* values only. Nominal *p* values and, where calculated, *q* values are distinguished in the tables. For each set we report the minimum detectable effect, that is, the odds ratio closest to 1 that the observed standard error would allow to be detected in a specified direction with 80% power at a two-sided significance level of 0.05. This describes what the data can resolve rather than being a conventional prospective power calculation, and we use that term in preference to power throughout [41]. Japanese exposure and European outcome cohorts are ancestrally distinct; overlap for Japanese outcomes requires cohort-level assessment [42]. The exposure scale, participant overlap, multiple-testing structure and limits of the MR-Egger intercept in this setting are described in the Supplementary Methods.

Carrying proxies discovered in Japanese cohorts into predominantly European outcome studies requires two assumptions. The first is that the per-allele effect of each variant on the serological phenotype is the same in both populations. The second is that each variant stands in the same relation to the surrounding haplotype in both, so that whatever else it tags is unchanged. Neither can be verified from summary statistics alone, and we therefore evaluated their plausibility: the first by looking the variants up in the largest European antibody dataset, and the second by comparing what they tag in European and East Asian reference panels. Because their validity is uncertain and the tagging patterns differ across ancestries, the estimates reported below should not be read as estimates of a causal effect of *H. pylori* infection. They are a demonstration that these instruments are unstable, and their direction and magnitude are of interest only insofar as these change with the composition of the proxy set, with the reference population and with the antibody trait substituted for *H. pylori*. The cross-ancestry comparisons reported below are therefore presented as an assessment of instrument validity and transferability between populations, and not as ancestry-specific estimates of a causal effect of infection.

### 2.5. Colocalisation

We tested whether the serological signal and each disease signal arose from the same variant using a standard Bayesian approach, centred on the strongest antibody variant, as recommended for genetic studies of infection serology [26,43]. Colocalisation requires summary statistics for variants across the region of interest for both traits. These have not been released for the Japanese discovery study, and we used publicly available region-wide antibody statistics measured in European participants [20], which is why the European antibody datasets were used here. For comparisons with European neurodegenerative outcomes, this reduces ancestry mismatch. The European antibody phenotype and sample differ from those of the Japanese discovery study, however, and these analyses do not directly test colocalisation of the Japanese discovery signal. We therefore treat them as supporting evidence under the stated model assumptions. Because conclusions can depend on the window and prior, we varied both (windows of 100, 250 and 500 kilobases; priors from 1 × 10^−7^ to 1 × 10^−5^), reported all five posterior probabilities for every combination, attempted to relax the single-causal-variant assumption by fine mapping, and applied the same analysis to peptic ulcer disease and its subtypes [44]. We distinguish throughout between a high posterior probability of two distinct causal variants, which is positive evidence against a shared signal, and a low probability of a shared variant that arises because one trait has no detectable signal in the region, which is not. Throughout, colocalisation results are described as supporting or not supporting a shared causal variant. Because the method rests on a specified model of the region, a high posterior probability for distinct causal variants is evidence for, rather than proof of, distinct causal mechanisms.

### 2.6. Linkage Disequilibrium Across Ancestries

Using 1000 Genomes reference data [45], we measured how strongly each proxy correlated with the leading disease variants and with established DR4 and DR15 haplotype markers, separately in European and East Asian samples, and whether the proxies remained mutually independent when moved between populations. The selection was held fixed and the same 22 variants were looked up regardless of their significance in European data (Supplementary Methods).

### 2.7. Attributing the Signal

Four analyses addressed the mechanism directly. We correlated each proxy with reported tag variants for classical class II alleles in both panels and looked up each tag’s association with every outcome. We repeated each disease association using approximate summary-statistic conditioning on the leading class II variant of that dataset, and the reverse [46]. We estimated how much a single class II signal accounted for by fitting a model in which each proxy affected disease only through its correlation with that variant, and repeated it under East Asian rather than European linkage disequilibrium. We corrected the discovery estimates for selection at genome-wide significance by conditional maximum likelihood. Derivations and limitations are in the Supplementary Methods.

### 2.8. Survey of Published Studies

We searched PubMed on 9 September 2026 using the strategy (“Mendelian randomization” OR “Mendelian randomisation”) AND (antibod*[tiab] OR IgG[tiab] OR serolog*[tiab] OR seropositiv*[tiab] OR seroreactiv*[tiab] OR infect*[tiab] OR pathogen*[tiab] OR virus*[tiab] OR viral[tiab] OR bacteri*[tiab]) AND (Parkinson*[tiab] OR Alzheimer*[tiab] OR dementia[tiab] OR “amyotrophic lateral sclerosis”[tiab] OR neurodegenerat*[tiab]), with no date or language restriction, which returned 333 records. PubMed was the only bibliographic database searched. One author screened titles and abstracts against three criteria: a two-sample Mendelian randomisation design; an exposure that was a serological, antibody or clinically ascertained infection phenotype; and an outcome that was Parkinson’s disease, Alzheimer’s disease or another dementia, or amyotrophic lateral sclerosis. Studies of the gut microbiome, of circulating inflammatory markers, and of infection as an outcome rather than an exposure were excluded, as were reviews. Reference lists of the included reports were screened for further studies. Twelve studies met the criteria and were read in full [7,8,9,10,11,12,13,14,15,16,17,18]; two further reports met them but could not be obtained in full text and were therefore not assessed. One study using protein rather than antibody exposures was read for comparison [19]. For each, we recorded every mention of the major histocompatibility complex, the HLA genes and chromosome 6, together with the significance threshold actually applied; numerical reporting of instrument strength was not separately assessed for every included study (Supplementary Materials Table S5).

### 2.9. Control and Sensitivity Analyses

As a positive control, we applied the same proxies, in the same sets, to peptic ulcer disease in a Japanese study matching the ancestry in which they were discovered, examined its gastric and duodenal subtypes, and tested the difference between estimates. We also looked the proxies up in multiple sclerosis; reversed the analysis, using disease-associated variants as proxies; repeated the analysis with 18 further pathogen-antibody traits measured in the same participants on the same platform; and looked up every proxy in the GWAS Catalog [20].

### 2.10. Software and Reporting

Analyses used R 4.6.1 with TwoSampleMR 0.7.9, MendelianRandomization 0.10.0, coloc 5.2.3, susieR 0.14.2, MR-PRESSO 1.0 and ieugwasr 1.1.0. Linkage disequilibrium was taken from the 1000 Genomes phase 3 European and East Asian reference panels. Package versions and reference panels are in Supplementary Materials Table S11. Reporting follows STROBE-MR, the Mendelian randomisation extension of STROBE, and the completed checklist is Supplementary Materials Table S12 [47].

## 3. Results

### 3.1. Localisation of the Association Within the MHC

Using all available proxies, higher genetically predicted anti-*H. pylori* IgG seropositivity was associated with lower risk of Alzheimer’s disease (OR 0.83, 95% CI 0.73 to 0.95), and the point estimate for Parkinson’s disease lay in the same direction without reaching conventional significance (0.83, 0.65 to 1.06). Read on its own, the Alzheimer’s disease estimate would be taken as evidence that *H. pylori* protects against the disease.

**Figure 1:**
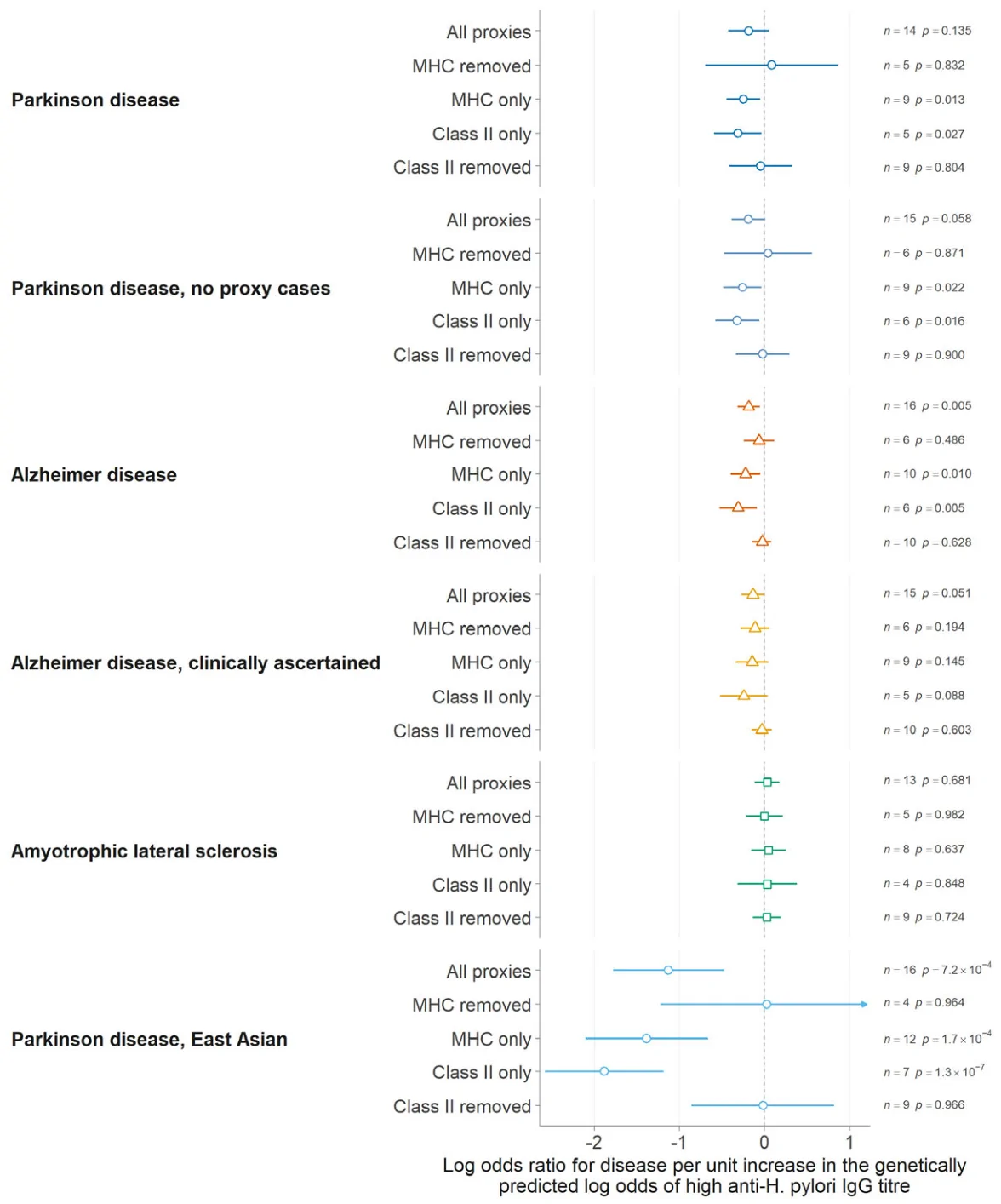
Associations with disease outcomes across genetically defined proxy sets. Log odds ratios express the association between each disease and a one-unit increase in the genetically predicted log odds of anti-*H. pylori* immunoglobulin G seropositivity. Associations were attenuated after variants in the major histocompatibility complex were removed and were strongest when analyses were restricted to human leukocyte antigen class II variants. The number of variants contributing to each estimate is shown.

The association was absent when MHC variants were removed (Alzheimer’s 0.94, 0.78 to 1.12; Parkinson’s 1.09, 0.50 to 2.37), strongest when only class II variants were used (0.73, 0.59 to 0.91 and 0.73, 0.55 to 0.97), and abolished when the class II variants were removed but the rest of the MHC variants were kept (0.97, 0.87 to 1.09 and 0.95, 0.66 to 1.38). Amyotrophic lateral sclerosis showed no association in any set (Figure 1, Table 2).

Instrument-strength statistics for the sets in Table 2 were between 41 and 72 in the discovery data; these values do not establish adequate instrument strength in European populations. For Alzheimer’s disease, heterogeneity was marked, and both the MR-Egger intercept and MR-PRESSO indicated that the proxies were not acting through one pathway, consistent with proxies tagging a haplotype; estimators designed to tolerate such heterogeneity did not differ materially (Supplementary Materials Table S2).

**Figure 2:**
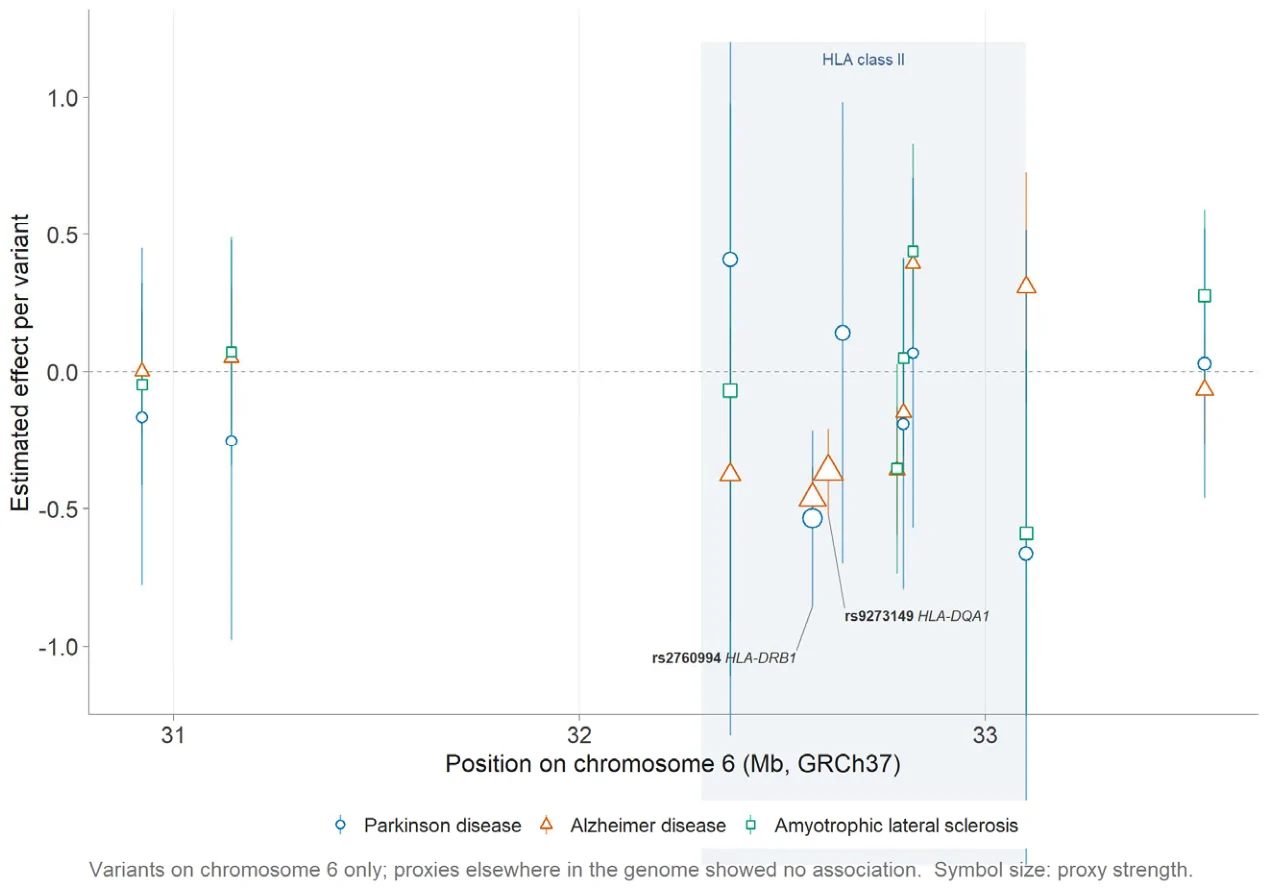
Variant-specific estimates across chromosome 6. Each point represents a variant-specific estimate, and symbol size is proportional to instrument strength. Estimates from two variants near *HLA-DRB1* and *HLA-DQA1* account for most of the association, whereas estimates for the remaining variants are centred near the null. In the Parkinson’s disease data, rs9273149 is absent unless a linkage-disequilibrium substitute is admitted, so only rs2760994 appears for that outcome.

The entire signal came from two variants, one near *HLA-DRB1* (rs2760994) and one near *HLA-DQA1* (rs9273149). Removing both left the class II estimate at 0.93 (0.66 to 1.32) for Alzheimer’s disease and 1.00 (0.68 to 1.46) for Parkinson’s disease; in the Parkinson’s disease data, rs9273149 is not present without substitution, so that estimate reflects the removal of rs2760994 alone (Figure 2, Supplementary Materials Table S3, Supplementary Materials Figure S1). Because deciding which to remove could itself introduce bias, we repeated this with a criterion fixed in advance, which is dropping any proxy correlated at *r*^2^ of 0.30 or more with any of five disease lead variants in the European reference panel; this selected three proxies, rs2760994, rs9273149 and rs28441428, and gave the same result. In East Asian Parkinson’s disease, the association instead persisted after the same removal (0.10, 0.04 to 0.30), a pattern consistent with the proxies tagging different neighbouring haplotypes across populations. That dataset contained only 340 cases, so the estimate indicates direction rather than magnitude.

Minimum detectable odds ratios, calculated from the observed standard errors at 80% power and a two-sided alpha of 0.05, were 0.77 for Alzheimer’s disease, 0.79 for clinically ascertained Alzheimer’s disease, 0.17 for East Asian Parkinson’s disease and 0.33 for European Parkinson’s disease using variants outside the MHC. These are sensitivity benchmarks, not formal tests of differences between proxy sets. In clinically ascertained Alzheimer’s disease, the class II estimate was itself imprecise (OR 0.78, *p* = 0.088), so it does not provide a confirmed association against which to interpret the non-MHC result. The European Parkinson’s disease benchmark is also insufficient to resolve an effect of the class II point estimate (OR 0.73; Supplementary Materials Table S6).

### 3.2. Positive Control: Peptic Ulcer Disease

If the class II proxies carry information about *H. pylori* infection, they should predict peptic ulcer disease. Applied to a Japanese study of 29,739 cases, matching the ancestry in which they were discovered, they did not: the class II estimate was 1.02 (0.92 to 1.13), and for the gastric and duodenal subtypes it was 1.02 (0.91 to 1.14) and 1.10 (0.95 to 1.28). This null is informative rather than imprecise: the minimum detectable effect for this set is an odds ratio of 1.16 or 0.86, so an effect of the magnitude seen in the neurodegenerative outcomes would have been detected with probability above 99%.

We compared outcome-specific estimates using an approximation that treats their estimation errors as independent. The exposure scale is common, but the available variants differ across outcomes; these comparisons therefore do not isolate outcome differences from instrument-composition differences. For Parkinson’s disease, the class II estimate was 0.73 against 1.02 for ulcer (difference *p* = 0.029); for Alzheimer’s disease, the estimate was 0.73 against 1.02 (*p* = 0.008). Nominal differences were also present in the all-variant and MHC-only sets (Supplementary Materials Table S10).

The peptic ulcer results weaken a simple interpretation of these class II variants as infection proxies, but do not establish that they contain no information about infection. Peptic ulcer disease includes non-*H. pylori* causes, and IgG titres reflect past as well as current infection. These features may attenuate an association. The ulcer results constrain associations with the measured ulcer phenotypes; they do not quantify an upper bound on how much information the proxies carry about infection (Supplementary Materials Table S6).

### 3.3. Colocalisation and Conditional Analysis

In the three *H. pylori* outer membrane protein (OMP) comparisons, the single-causal-variant model favoured distinct causal variants over a shared variant. At the primary setting, a 250-kilobase window with a prior of 1 × 10^−6^, the posterior probability of distinct causal variants was 0.993 for Alzheimer’s disease, with the remainder distributed as 0.007 for a signal in the disease trait alone, 4.1 × 10^−5^ for a shared causal variant, 8.1 × 10^−14^ for a signal in the antibody trait alone and 5.6 × 10^−16^ for no signal in either. The corresponding five probabilities were 0.980, 0.020, 4.6 × 10^−5^, 4.1 × 10^−6^ and 8.3 × 10^−8^ for Parkinson’s disease, and 0.975, 0.025, 7.4 × 10^−5^, 2.7 × 10^−6^ and 6.7 × 10^−8^ for amyotrophic lateral sclerosis. The probability of a shared causal variant remained below 0.002 across the 108 antibody-by-outcome-by-window-by-prior analyses, including 36 *H. pylori* OMP analyses (Supplementary Materials Table S8). Because both traits carry a detectable signal in the region in each of these comparisons, this pattern supports separate causal variants rather than reflecting an absence of evidence for a shared one. Under the assumptions of the method, these posterior probabilities suggest, but do not prove, that the serological and disease signals arise from different causal variants. The ulcer comparisons differed by subtype: at the 250-kilobase, 1 × 10^−6^ setting, the posterior probability of distinct causal variants (PP.H3) was 0.765 for peptic ulcer disease, 0.993 for duodenal ulcer and 0.359 for gastric ulcer; gastric ulcer instead had a posterior probability of 0.636 for a signal in the antibody trait alone (PP.H1). These results should not all be described as an absence of a disease signal. We do not use them as decisive mechanistic evidence, given their varying support across hypotheses and the ancestry mismatch between European antibody and Japanese ulcer data.

Visual inspection is not sufficient here: for Alzheimer’s disease, the leading variants of the two traits lie 2 kilobases apart and appear to coincide, yet the formal analysis still favours distinct causal variants there (Supplementary Materials Figure S2).

Approximate conditional analysis provides complementary evidence about dependence on the leading disease variants. Conditioning the Alzheimer’s disease association of the *HLA-DQA1* proxy on the leading class II variant attenuated it below nominal significance (from *p* = 5.9 × 10^−6^ to *p* = 0.27), and the Parkinson’s disease association of the *HLA-DRB1* proxy likewise (from 1.1 × 10^−3^ to 0.44). In both, the leading variant kept its own association after conditioning on the proxy (2.0 × 10^−19^ to 2.1 × 10^−12^; 4.1 × 10^−11^ to 2.4 × 10^−9^). For Alzheimer’s disease, the *HLA-DRB1* proxy, only weakly correlated with that lead (*r*^2^ = 0.06), was attenuated but not abolished (8.0 × 10^−16^ to 1.1 × 10^−8^), consistent with tagging a different haplotype. In peptic ulcer disease, both proxies were null before and after (Supplementary Materials Table S16).

### 3.4. Cross-Ancestry Haplotype Tagging and Instrument Transferability

**Figure 3:**
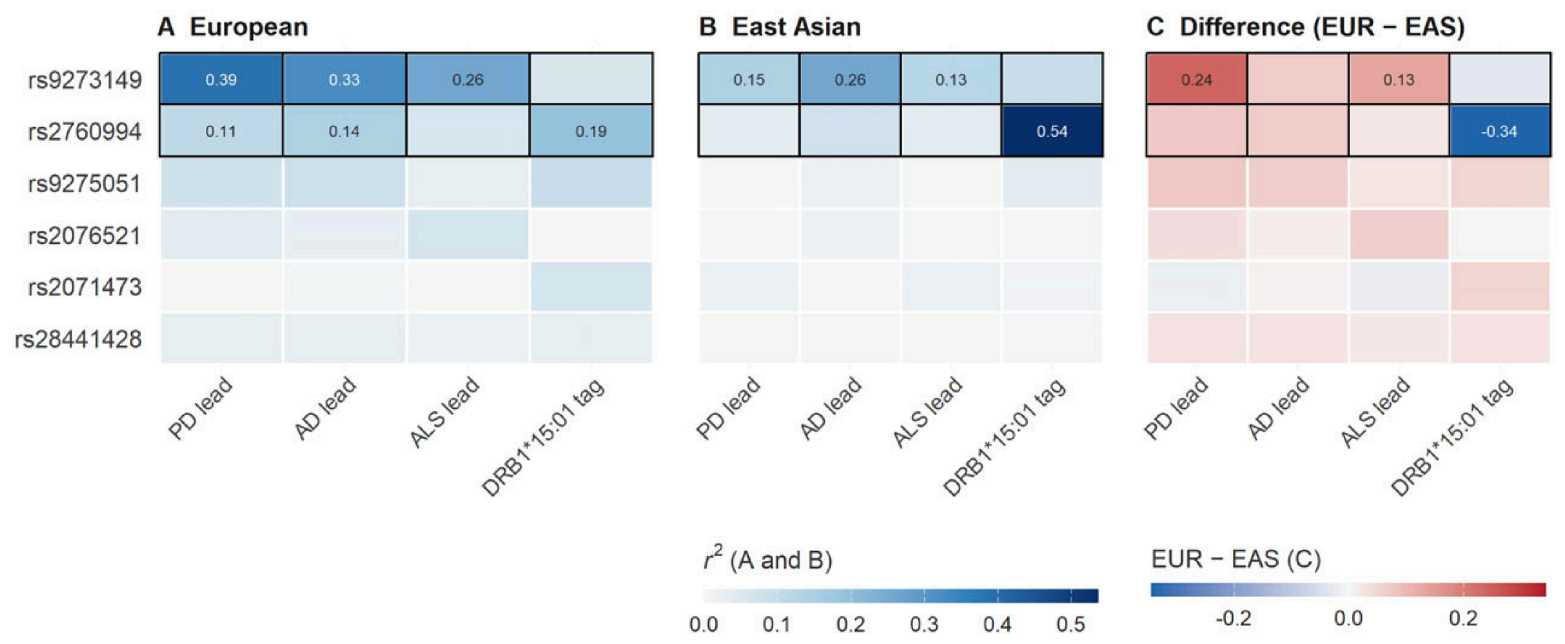
Linkage disequilibrium between antibody-response proxies and leading disease variants by ancestry. Squared correlation coefficients were estimated in European and East Asian reference samples, shown in (**A**) and (**B**), respectively, with the difference between ancestries in (**C**). Values below 0.10 are not labelled. Outlined cells identify the two variants that contributed most strongly to the association and show that their linkage disequilibrium patterns differ between populations.

These comparisons ask whether the proxies retain their meaning when they are moved between populations, and they are reported as evidence on instrument transferability rather than as ancestry-specific causal estimates. The two proxies that carried the signal behaved in opposite ways in the two populations (Figure 3). The variant near *HLA-DQA1* correlated closely with the leading Parkinson’s disease variant in Europeans (*r*^2^ = 0.39) but much less in East Asians (0.15), and the same pattern held across all seven DR4 markers; the variant near *HLA-DRB1* showed the reverse for DR15, with three of six markers being substantially more strongly correlated in East Asians (0.35 to 0.73, against 0.09 to 0.21) (Supplementary Materials Table S4). In the Japanese population, one proxy therefore tags DR15 while predicting seropositivity, whereas in European data the other overlaps a protective HLA-DRB1*04-associated signal for Parkinson’s and Alzheimer’s disease. This structure can generate an apparently protective estimate without requiring a causal effect of *H. pylori* infection.

The same relationship can be expressed in terms of classical alleles. The variant near *HLA-DRB1* correlates with the reported tag for HLA-DRB1*15:01 at *r*^2^ = 0.19 in Europeans and 0.54 in East Asians, and with a second DR2 marker at 0.21 and 0.73; the variant near *HLA-DQA1* moves the other way, correlating with the tag for HLA-DRB1*04:01 at 0.26 against 0.09. The tags behave as predicted: the tag for HLA-DRB1*04:01 is associated with Alzheimer’s disease at *p* = 2.4 × 10^−12^ and Parkinson’s disease at *p* = 5.8 × 10^−7^ but not peptic ulcer disease (*p* = 0.34), whereas the tag for HLA-DRB1*15:01 was associated with Alzheimer’s disease but not Parkinson’s disease or peptic ulcer disease (*p* = 8.9 × 10^−5^, 0.36, 0.22; Supplementary Materials Table S15).

An exploratory one-parameter model of linkage to the leading class II variant explained 64% of the variation in the Alzheimer’s disease proxy associations (Supplementary Materials Table S17; Supplementary Materials Figure S3). Residual heterogeneity remained significant (*p* = 2 × 10^−7^), so one signal was not sufficient. The listed Parkinson’s disease and peptic ulcer disease inputs differ from the current primary instrument sets; their model fits and ancestry-transfer ratios therefore do not quantify how much of the current primary associations is explained. These comparisons require reanalysis with matched variants and allele coding.

The proxies are also unstable in a more basic respect. The 22 proxies were selected to be mutually independent in the Japanese sample, at *r*^2^ below 0.05; in European reference data, ten pairs exceed that threshold, reaching 0.20. This too is confined to the MHC, the largest pairwise correlation being 0.29 within the region and 0.007 outside. Repeating each estimate with a weighting scheme that incorporates these correlations, using a reference panel matched to the ancestry of each outcome and restricted to the variants that panel contains, changed the standard errors by factors of 0.92 to 1.17 and left the direction of every estimate unchanged. For the class II sets, which carry the argument, no contributing variant was missing from the panel, so those comparisons are made on the same variants as the primary analysis (Supplementary Materials Table S2).

We then examined whether the variants function as proxies for the exposure in Europeans. Of the 17 present in the largest European antibody dataset, effects agreed in direction with the Japanese study in 12 and correlated only moderately (*r* = 0.44); none reached the conventional threshold for a usable instrument, the largest *F* statistic being 7.3 and all others between 0.0 and 3.5, against 28 to 146 where they were discovered. These variants did not show adequate strength in this European antibody dataset. Differences in sample size and antibody phenotype also contribute to this comparison, so it does not by itself establish a loss of per-allele effects across ancestries. Taken together, the ancestry-dependent tagging and the weak associations in the European antibody data indicate that these instruments are unstable and transfer poorly from the Japanese discovery population to European samples. That instability, rather than any ancestry-specific effect of infection, is the inference we draw from the cross-ancestry analyses.

### 3.5. Other Pathogen-Antibody Traits

**Figure 4:**
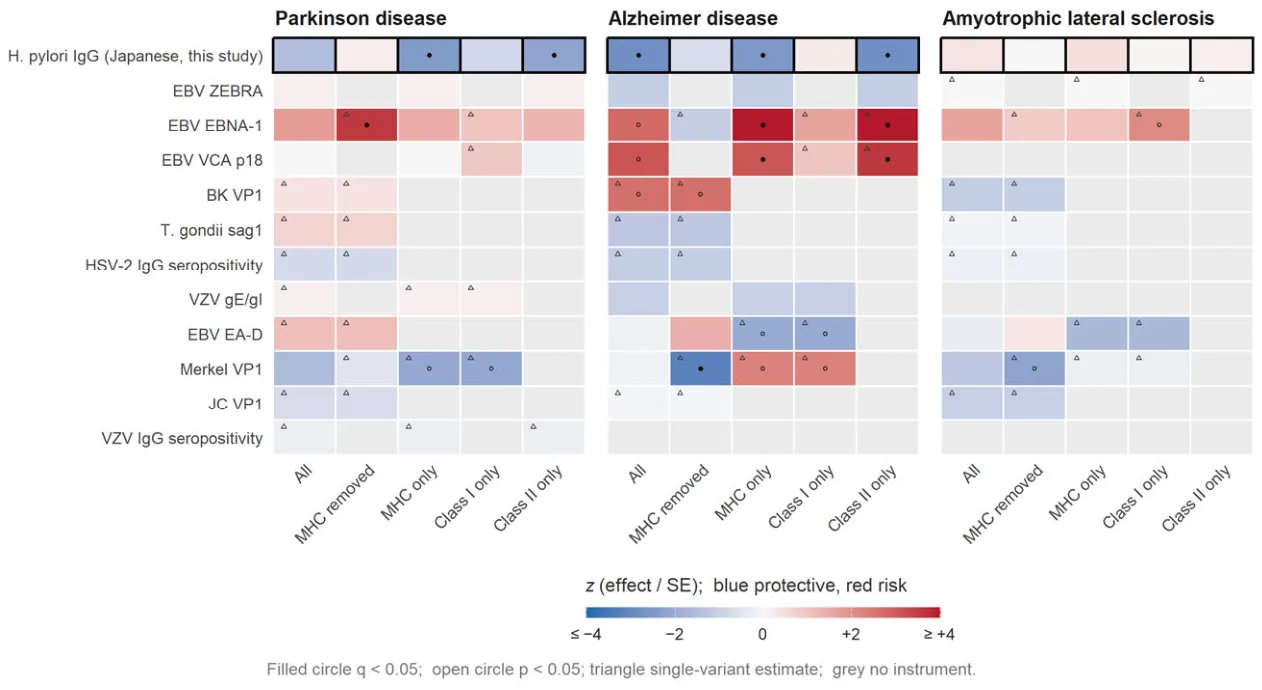
Associations of 12 pathogen-antibody traits with neurodegenerative disease outcomes. The additional pathogen-antibody traits were measured in the same European study. The Japanese *H. pylori* IgG instrument is a separate benchmark; all traits were analysed using the same five genomic proxy-set definitions. Cell shading represents the effect estimate divided by its standard error, allowing comparison across traits measured on different scales. Filled circles indicate associations that remained significant after correction for multiple testing, open circles indicate nominal significance, and triangles indicate single-variant estimates. Grey cells indicate that no genetic instrument was available. The anti-*H. pylori* immunoglobulin G trait is outlined.

We took 18 further antibody traits measured in the same participants on the same platform, of which 11 yielded proxies at conventional thresholds, and repeated the analysis with each as the exposure (Figure 4; Supplementary Materials Table S5). Most yielded no class II proxies, so only four exposures could be compared with *H. pylori* in that set for Alzheimer’s disease and five for Parkinson’s disease, but within those few the pattern was clear. Epstein–Barr nuclear antigen 1 antibodies gave an Alzheimer’s disease estimate of 1.15 (*p* = 3.5 × 10^−8^) and viral capsid antigen p18 antibodies 1.18 (*p* = 3.1 × 10^−4^), both opposite in direction to the 0.73 for *H. pylori*. Associations differed across antibody traits, but the comparison with the Japanese *H. pylori* IgG instrument also changes the exposure population and assay, so that contrast cannot be attributed to antibody specificity alone.

### 3.6. Association with Multiple Sclerosis

Multiple sclerosis has the best characterised class II association of any disease and therefore provides a direct test of what these variants predict. Of the 17 proxies present in that consortium meta-analysis, the variant near *HLA-DRB1* was associated at *p* = 8.8 × 10^−120^, a second class II proxy, near *HLA-DQA1*, was associated at *p* = 1.6 × 10^−104^ and a third, near *HLA-DOB*, was associated at *p* = 7.8 × 10^−41^ (Supplementary Materials Table S9).

**Figure 5:**
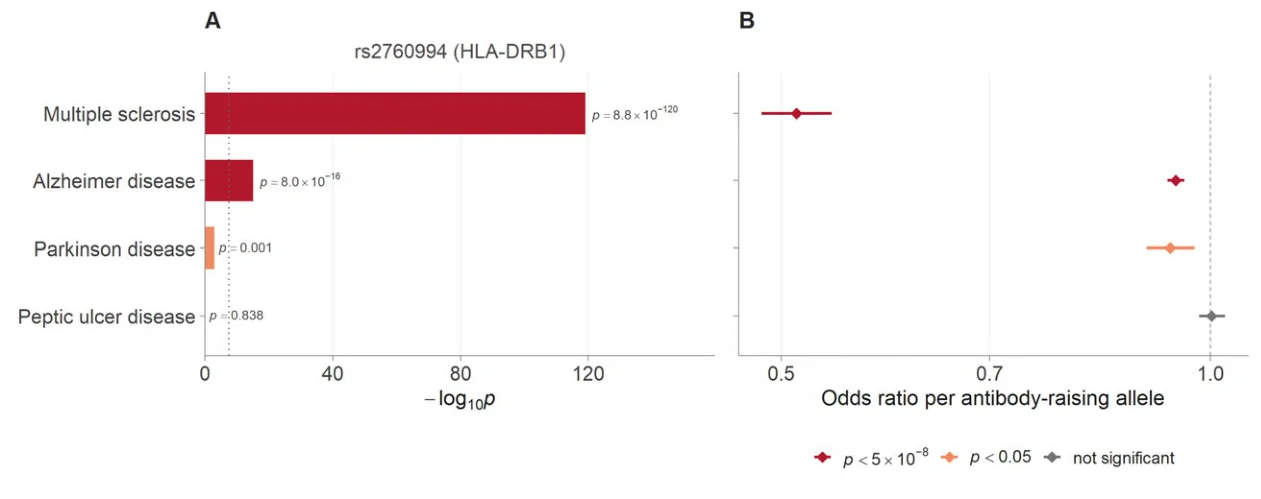
Disease associations of rs2760994. The rs2760994 variant near *HLA-DRB1* accounts for much of the reported association. Results are shown for multiple sclerosis, for which human leukocyte antigen class II variation is a major genetic risk factor; Alzheimer’s and Parkinson’s disease, for which class II effects are weaker; and peptic ulcer disease, which is causally related to *H. pylori* infection. The displayed allele is associated with a higher probability of anti-*H. pylori* immunoglobulin G seropositivity. The strength of statistical evidence is shown in (**A**), and odds ratios with 95% confidence intervals in (**B**). Amyotrophic lateral sclerosis is omitted because this variant was unavailable in that dataset.

Comparing the four outcomes for this variant uses marginal associations for the same allele in each disease and avoids Mendelian randomisation ratio modelling, taken from the dataset used elsewhere here (Figure 5). It is associated with multiple sclerosis at *p* = 8.8 × 10^−120^ (0.51, 0.48 to 0.54), Alzheimer’s disease at *p* = 8.0 × 10^−16^ (0.95, 0.93 to 0.96), Parkinson’s disease at *p* = 0.001 (0.94, 0.90 to 0.97), and peptic ulcer disease at *p* = 0.84 (1.00, 0.98 to 1.02). The strong multiple sclerosis association is compatible with class II tagging. Cross-disease *p* values also depend on sample size, precision and phenotype definition, so their ordering alone does not distinguish infection effects from haplotype tagging.

Amyotrophic lateral sclerosis behaves in the opposite way: it has a class II association of its own, yet these proxies showed none with it in any set (Table 2), indicating that they track a particular haplotype rather than the region as a whole. Two features of the multiple sclerosis data limit what can be drawn from them: 75% of class II variants there reach genome-wide significance, so we report single-variant associations rather than a pooled estimate; the proxies outside the MHC were themselves associated with multiple sclerosis (1.53, 1.15 to 2.03).

Reverse Mendelian randomisation produced no association when 22 proxies for Parkinson’s disease (−0.02, −0.12 to 0.09) or 64 for multiple sclerosis (−0.00, −0.06 to 0.05) were applied to the serological phenotype (Supplementary Materials Table S14). These analyses provided no evidence of reverse-direction effects; they neither exclude such effects nor distinguish a causal exposure-to-disease effect from horizontal pleiotropy.

### 3.7. Sensitivity Analyses

The GWAS Catalog returned 22 genome-wide significant associations across the 22 variants. Only rs2760994 was associated with a phenotype conventionally treated as a confounder here, namely lipid measurements; none were associated with smoking, adiposity, alcohol or educational attainment. Six of the 16 proxies inside the MHC had reported associations against one of the six outside, and the traits are characteristic of the region. rs2760994 is itself associated with Epstein–Barr virus seropositivity as well as with neurodegenerative disease, a pattern expected of a marker of antigen presentation (Supplementary Materials Table S13).

Several additional sensitivity analyses are tabulated in Supplementary Materials Tables S2, S3 and S18. Correcting the discovery coefficients for selection at genome-wide significance moved the class II estimate further from the null for Alzheimer’s disease (0.73 to 0.67) and for European Parkinson’s disease (0.73 to 0.67), so the winner’s curse attenuates rather than generates the associations reported in the European datasets. The East Asian Parkinson’s disease estimate moved in the opposite direction, from 0.15 to 0.28, and lost nominal significance, and the sets restricted to class I contain variants selected close to the threshold whose corrected estimates are unstable. These estimates use the instrument sets and the variance rule of the primary analysis (Supplementary Materials Table S18). Variant-specific estimates plotted against precision show the negative side of the Alzheimer’s disease plot being occupied largely by class II variants (Supplementary Materials Figure S4).

## 4. Discussion

### 4.1. Principal Findings

The reported genetic association between anti-*H. pylori* IgG seropositivity and neurodegenerative disease was confined to HLA class II and driven by two variants. Taken together, the evidence is more consistent with haplotype tagging than with a causal effect of the measured serological phenotype: the variants did not predict ancestry-matched peptic ulcer disease, colocalisation did not support a shared causal variant for the serological and disease signals, selected proxy associations attenuated after conditioning on leading disease variants, and the pattern changed with the ancestry and pathogen-antibody trait. The conclusion therefore does not depend on any single Mendelian randomisation estimator or sensitivity analysis. We would not claim that the two explanations have been separated completely. HLA class II variation may plausibly influence antibody production, susceptibility to infection, its persistence and its clearance at the same time, so a variant can carry information about antigen presentation and about infection together. The analyses support a substantial contribution from class II haplotype structure, but do not show that one HLA signal fully explains the associations: the exploratory one-parameter model explained 64% of proxy-disease variation for Alzheimer’s disease, with significant residual heterogeneity. Its Parkinson’s disease input set requires updating before a corresponding proportion can be stated for the revised analysis. These findings undermine a causal interpretation of the serological instruments.

The comparison in Figure 5 depends on the fewest modelling assumptions: one variant, one allele, and the association reported directly by each disease dataset. The much stronger association with multiple sclerosis is compatible with HLA tagging, although a valid infection proxy is not required to produce the smallest *p* value for peptic ulcer disease. Two independent meta-analyses found no association between *H. pylori* infection and multiple sclerosis, with pooled odds ratios of 0.79 (0.58 to 1.08) [48] and 0.81 (0.59 to 1.10) [3], yet multiple sclerosis was the outcome with which this proxy was most strongly associated. This comparison is descriptive and should be interpreted with differences in outcome precision and ascertainment in mind.

**Figure 6:**
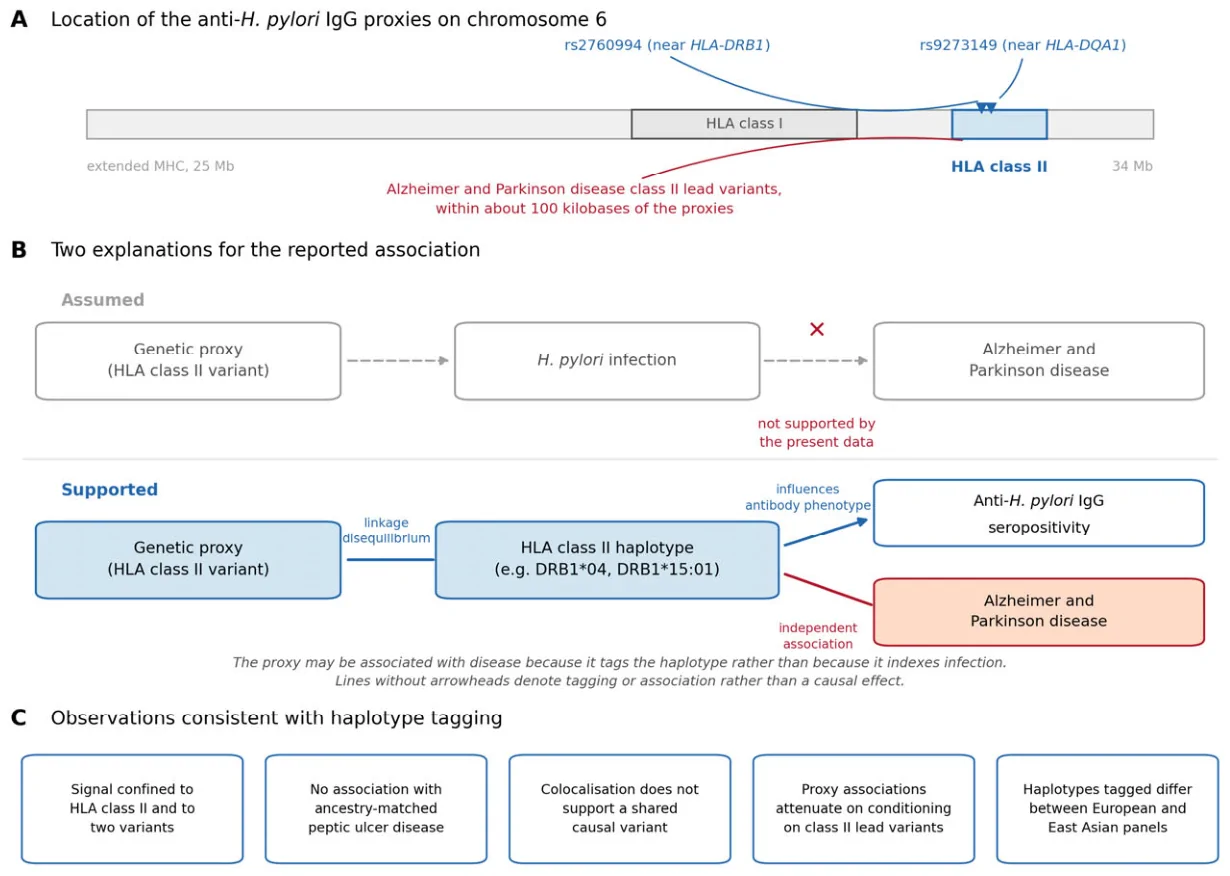
Proposed mechanism by which the reported proxies capture HLA class II haplotype structure rather than liability to *H. pylori* infection. (**A**) The candidate proxies for anti-*H. pylori* immunoglobulin G seropositivity lie almost entirely within the extended major histocompatibility complex, and the two variants that carry the association, rs2760994 near *HLA-DRB1* and rs9273149 near *HLA-DQA1*, fall within human leukocyte antigen class II, within about 100 kilobases of the class II lead variants for Alzheimer’s and Parkinson’s disease. (**B**) Under the assumed model, the proxies influence disease only through infection. Under the model supported by the present analyses, the proxies may tag class II allele or haplotype backgrounds, such as those carrying HLA-DRB1*04 or HLA-DRB1*15:01, that influence the antibody phenotype and are independently associated with disease, so the assumption that the proxies affect disease only through the exposure would not hold. Arrows denote hypothesised causal effects, and lines without arrowheads denote tagging or statistical association rather than a causal effect. (**C**) Observations from the present study that are consistent with the second model. The figure is a schematic summary and is not drawn to scale.

The cross-ancestry comparisons carry a specific and limited meaning within this argument. They show that the proxies do not keep their relation to the surrounding haplotype, nor their strength as predictors of the serological phenotype, when they are moved from the Japanese discovery population to European samples. What they demonstrate is the instability and limited transferability of these instruments rather than an ancestry-specific biological effect of infection, and the estimates obtained in European outcome datasets should be read in that light. Figure 6 summarises the mechanism we propose: variants selected as proxies for anti-*H. pylori* immunoglobulin G seropositivity lie on HLA class II haplotypes that are themselves associated with Alzheimer’s and Parkinson’s disease, so an apparently protective association can arise without any effect of infection.

### 4.2. Implications for Research on H. pylori

Our Alzheimer’s disease estimate, which concerns genetically predicted IgG seropositivity rather than measured infection, points opposite to the observational literature on infection, although that evidence is weaker than the headline figures suggest: the pooled interval barely excludes the null (1.43, 1.01 to 2.02) [3], and the prospective study that measured titres directly found no association over 22 years [49]. For Parkinson’s disease, the observational evidence is stronger, and our findings indicate that it is not supported by the available genetic proxies. Studies from our institution are among those reporting positive findings, including cortical thinning in infected men without cognitive impairment [50] and improvement in levodopa on-time after eradication [5]. Neither is in direct tension with the present analysis: one concerns an imaging phenotype and the other a pharmacokinetic effect in established disease.

These findings are narrow in scope. They concern the genetic evidence linking *H. pylori* serology to neurodegenerative disease and say nothing about the organism’s established role in the stomach. What they show is that eradication cannot at present be justified on genetic grounds as a means of preventing neurodegenerative disease. This does not bear on the accepted clinical indications for eradication. Treatment of *H. pylori* in peptic ulcer disease, in gastric mucosa-associated lymphoid tissue lymphoma, after endoscopic resection of early gastric cancer, and for the prevention of gastric cancer rests on randomised and observational evidence of its own [2], and nothing here questions it. That is also a different question from whether treating established Parkinson’s disease helps, though the two sit comfortably together: a placebo-controlled trial found no improvement in motor, non-motor or quality-of-life outcomes after eradication [6]. A Mendelian randomisation estimate concerns lifelong liability to seropositivity, whereas a trial changes infection status once, in adults.

### 4.3. Implications for Mendelian Randomisation Studies of Infection Serology

The same mechanism explains both the large ratio estimates and the conflicting conclusions of studies using this antibody panel [7,8]. The proxies have modest effects on seropositivity but overlap disease variants with substantially larger effects, so small changes in proxy selection can produce large and unstable ratio estimates. At conventional thresholds these traits yield few variants, almost all in the MHC; relaxing the threshold, as several studies have done [7,8,12,17], increases the number roughly fifteen-fold while reducing the proportion in the MHC to under 5% (Supplementary Materials Table S5). One study reported a causal effect of varicella zoster antibodies on Parkinson’s disease without mentioning the MHC, although every genome-wide significant variant for that trait lies on chromosome 6 [13]. The same panel and design have produced reported causal effects for Epstein–Barr virus [12], human papillomavirus [9], herpes simplex virus 1 [11], sepsis [14] and hospital-treated infection [15], whereas analyses of SARS-CoV-2 [10] and herpes viruses in amyotrophic lateral sclerosis [16] found none.

The concern may extend beyond neurodegeneration. A bidirectional analysis of the same antibody traits reported that antibodies to the outer membrane protein increased the risk of Graves’ disease (1.15, 1.09 to 1.22) [51], but the only genome-wide significant variant for that antibody trait in Europeans lies within *HLA-DQB1*. Wherever the MHC goes unexamined, a serological proxy built on it will tend to recover the HLA association of whatever autoimmune disease it is pointed at.

Five checks would each have caught this, and we suggest them for Mendelian randomisation studies of infection serology [26]: report results with and without the MHC; show that proxies retain their meaning across ancestries; include an ancestry-matched outcome that the pathogen is known to cause; repeat the analysis with unrelated pathogen-antibody traits measured on the same platform; and report proxy strength numerically.

### 4.4. Strengths and Limitations

Several features of the design support the inference. The analyses that converge on it rest on different assumptions, so a flaw in any one of them leaves the others standing: removing the region, locating the signal within it, testing an ancestry-matched disease the organism is known to cause, asking whether the two signals share a causal variant, conditioning on the leading disease variant, and comparing what the proxies tag in two populations. The five proxy sets were fixed before any estimate was seen and applied identically to every exposure and outcome, which limits the room for choosing an informative subset afterwards. The peptic ulcer comparison asks whether the variants behave as markers of the organism in the population where they were found, and the European serology and cross-ancestry comparisons ask whether they survive being moved. None of this rules out every biological effect of *H. pylori* infection; what it constrains is the interpretation of these particular genetic instruments.

Against this, several things limit what we can claim. Full summary statistics for the exposure have not been released, so we used reported values for lead variants, and the assignment of classical HLA alleles warrants separate scrutiny. The discovery phenotype uses dataset-specific cut-offs (the top quartile or 3 U/mL), rather than one uniform clinical seropositivity definition, so the exposure is liability to a high antibody response, which stands a further step from infection status. Anti-*H. pylori* IgG serology cannot distinguish current from past infection [2], and a dichotomised assay phenotype yields an estimate for liability underlying seropositivity rather than the effect of changing infection status [21]. We applied proxies derived in a Japanese population to European outcomes, which is both the subject of this paper and a limitation, and the tag variants represent classical alleles imperfectly and differently across populations. Cross-ancestry tagging differences and weak associations in the European antibody data raise concerns about transportability; the ratio estimates should therefore not be interpreted as causal effects of infection. Peptic ulcer disease is an imperfect positive control because its case definition includes non-*H. pylori* causes, including drug-related and idiopathic ulcers; that misclassification would tend to blunt an association with infection.

The primary colocalisation method assumes a single causal variant, which is a strong assumption in a region containing several. We attempted to relax it [44], but no credible set could be identified for the *H. pylori* trait, whose sample size was too small to localise a signal in this region; the fine-mapping analysis is therefore uninformative, and the colocalisation result rests on the single-variant model. We regard it as consistent with the rest of the evidence rather than as establishing the point on its own. Accordingly, we describe these analyses as supporting distinct causal variants for the serological and disease signals, and not as proving them. For multiple sclerosis, we report single-variant associations rather than a pooled estimate because heterogeneity exceeded 99% and the direction changed with the proxies included. These constraints limit the precision of individual mechanistic assignments but not the central observation that the reported association is concentrated in HLA class II.

## 5. Conclusions

In conclusion, the reported inverse genetic associations of anti-*H. pylori* IgG seropositivity with Parkinson’s and Alzheimer’s diseases do not provide evidence that *H. pylori* infection protects against neurodegeneration. The associations were concentrated in HLA class II and attenuated after removal of two influential variants; selected proxy associations also attenuated in the reported approximate conditional analyses; and together with the absence of association with ancestry-matched peptic ulcer disease, the lack of colocalisation support for a shared causal variant, and ancestry-dependent linkage disequilibrium indicating limited transferability of the instruments, this makes HLA class II haplotype tagging the most economical explanation, although it does not exclude the possibility that class II variation shapes susceptibility to infection and antibody response together. What follows for practice is narrower than the original reports implied: the currently available serological instruments are not suitable for inferring a preventive effect of infection, or for supporting *H. pylori* eradication as a strategy against neurodegenerative disease. Future Mendelian randomisation studies of infection serology should evaluate major histocompatibility complex dependence and ancestry-specific instrument validity before drawing causal or clinical conclusions.

## Figures and Tables

**Table 2:** Association estimates and diagnostic statistics by proxy set.

Outcome	Proxy Set	*n*	Odds Ratio (95% CI)	*p*	*q*	Mean *F*	Cochran Q (*p*)
Parkinson’s disease	All variants	14	0.83 (0.65 to 1.06)	0.135	0.162	55	24.5 (0.03)
	MHC excluded	5	1.09 (0.50 to 2.37)	0.832	0.982	58	15.1 (0.004)
	MHC variants only	9	0.78 (0.64 to 0.95)	0.013	0.026	53	7.2 (0.52)
	HLA class II variants only	5	0.73 (0.55 to 0.97)	0.027	0.041	64	5.1 (0.27)
Alzheimer’s disease	All variants	16	0.83 (0.73 to 0.95)	0.005	0.016	58	81.5 (<0.001)
	MHC excluded	6	0.94 (0.78 to 1.12)	0.486	0.982	55	11.8 (0.04)
	MHC variants only	10	0.80 (0.67 to 0.95)	0.010	0.026	59	64.2 (<0.001)
	HLA class II variants only	6	0.73 (0.59 to 0.91)	0.005	0.016	72	42.3 (<0.001)
Amyotrophic lateral sclerosis	All variants	13	1.03 (0.89 to 1.19)	0.681	0.681	48	19.0 (0.09)
	MHC excluded	5	1.00 (0.80 to 1.24)	0.982	0.982	60	5.0 (0.29)
	MHC variants only	8	1.05 (0.86 to 1.29)	0.637	0.637	41	13.9 (0.05)
	HLA class II variants only	4	1.03 (0.73 to 1.47)	0.848	0.848	41	8.1 (0.04)

Odds ratios are expressed per unit increase in the genetically predicted log odds of anti-*H. pylori* immunoglobulin G seropositivity. The *q* value controls the false discovery rate within each proxy set, the mean *F* is the arithmetic mean of the per-variant *F* statistics of the contributing variants, and *n* is the number of variants. Exposure-variance measures and instrument-strength statistics are reported in Supplementary Materials Tables S6 and S7. The MR-Egger intercept, MR-PRESSO global test and additional estimators are reported in Supplementary Materials Table S2. CI denotes confidence interval; HLA, human leukocyte antigen; and MHC, major histocompatibility complex.

## Data Availability

All datasets analysed during this study are listed with their accession numbers in Table 1. All are publicly available from the GWAS Catalogue (https://www.ebi.ac.uk/gwas/, accessed on 27 September 2026) or the OpenGWAS database (https://gwas.mrcieu.ac.uk/, accessed on 27 September 2026) except the multiple sclerosis summary statistics, which are available from the International Multiple Sclerosis Genetics Consortium on application. No individual-level data were used.

## Supplementary Materials

The following supporting information can be downloaded at https://www.mdpi.com/article/10.3390/microorganisms14102193/s1, Supplementary Information containing the Supplementary Methods and Supplementary Figures S1–S4, and Supplementary Tables S1–S20 (Excel workbook). Figure S1: Effect of removing the two variants that account for most of the association; Figure S2: Regional association patterns for the antibody response and disease outcomes; Figure S3: Fit of a one-parameter human leukocyte antigen class II model; Figure S4: Variant-specific estimates according to inverse standard error; Table S1: Genetic instruments for the anti-H. pylori antibody response; Table S2: Association estimates and pleiotropy diagnostics; Table S3: Influence of individual variants; Table S4: Linkage disequilibrium by ancestry; Table S5: Published studies, instrument-selection thresholds and pathogen-antibody traits; Table S6: Minimum detectable effects; Table S7: Harmonisation and variant exclusions; Table S8: Colocalisation analyses; Table S9: Multiple sclerosis analyses; Table S10: Key variants across outcomes; Table S11: Software and reference data; Table S12: STROBE-MR reporting checklist; Table S13: Previously reported associations of the proxy variants; Table S14: Reverse-direction analyses; Table S15: Attribution of classical human leukocyte antigen class II alleles; Table S16: Approximate conditional analyses; Table S17: Proportion of the association explained by a single class II signal; Table S18: Correction for winner’s curse; Table S19: Effect of linkage-disequilibrium proxy substitution on the Parkinson’s disease analyses; Table S20: Harmonisation audit for every candidate variant in every outcome dataset.

## Abbreviations

CI, confidence interval; GWAS, genome-wide association study; HLA, human leukocyte antigen; IgG, immunoglobulin G; MHC, major histocompatibility complex; MR, Mendelian randomisation; OMP, outer membrane protein; OR, odds ratio.
