# Supplementary material for: Genetic Proxies for Helicobacter pylori Serology Are Dominated by HLA Class II Haplotype Structure: Implications for Reported Associations with Neurodegenerative Disease
Source: Microorganisms. 2026 Oct 1;14(10):2193. doi: 10.3390/microorganisms14102193 (PMC13707645; doi:10.3390/microorganisms14102193)
Supplement: Supplementary file 1 [file microorganisms-14-02193-s001.zip › Supplementary_Information.pdf]

Supplementary Information

Genetic Proxies for Helicobacter pylori Serology Are Dominated by HLA Class II Haplotype Structure: Implications for Reported Associations with Neurodegenerative Disease

Hyuk Lee, Yang Won Min, Tae Jun Kim

Supplementary Tables S1-S20 are provided in the accompanying Excel workbook, Supplementary_Tables_S1-S20_v7.xlsx.

Supplementary Methods

Harmonisation, exclusions and genome builds

Effect alleles were aligned between the exposure and each outcome before analysis. Variants with complementary alleles, for which strand cannot be determined from the alleles alone, were resolved by comparing allele frequencies where these were unambiguous, and were excluded where the frequency was close to one half or where the two datasets disagreed. Harmonisation was performed separately for each outcome, and the number retained therefore varied across datasets.

Allele information was incomplete for several variants in the source publication; we recovered it where possible by matching against the outcome datasets. Genomic positions were confirmed against dbSNP, and two positions reported in the source publication were found to be incorrect and were corrected before analysis. The variants excluded at each stage, with the reason for exclusion, and the number of proxies surviving harmonisation for each outcome, are listed in Supplementary Table S7. Allele and position audits are given in the same table.

The two Alzheimer disease outcome datasets are reported on GRCh38 while the remainder are on GRCh37. Rather than lifting coordinates over, we matched on identifier where available and otherwise applied the constant offset of 32,223 base pairs that holds across the region examined, which we verified against variants present in both builds.

The primary Parkinson disease and East Asian Parkinson disease analyses were repeated with automatic linkage-disequilibrium proxy substitution disabled. Supplementary Table S19 compares estimates with and without substitution. This use of proxy variants is distinct from the inclusion of proxy cases identified through family history in a disease GWAS.

Exposure scale and effect measure

The exposure in the discovery study is anti-*H. pylori* IgG antibody titre, measured by enzyme-linked immunosorbent assay (E-Plate Eiken *H. pylori* antibody, Eiken Chemical) or by latex agglutination turbidimetry (*H. pylori*-latex Seiken, Denka Seiken) and dichotomised, because the titre distribution is strongly skewed, at a cohort-specific cut-off, either the top quartile of the titre distribution or 3 U/mL. The combined meta-analysis of six Japanese cohorts and fourteen datasets, which is the analysis from which the instruments are taken, compared 37,410 participants above that cut-off with 87,768 below it. The figures 58,106 and 67,072 belong to a sensitivity analysis in which a single 3 U/mL threshold was applied to every cohort; the source reports them once in its Results as though they described the main analysis, but its Methods, Table 1 and figure legends consistently give 37,410 and 87,768, and we follow those. The reported per-allele effects are therefore on the log odds scale of a high antibody titre rather than of a continuous titre, and rather than of a clinically defined seropositive result. We use seropositivity as shorthand for a titre above this cut-off throughout, and note that the exposure is liability to a high antibody response, which stands one step further from infection status than a clinical seropositivity phenotype would. Estimates in this paper are therefore expressed per unit increase in genetically predicted log odds of seropositivity, not per standard deviation, and we avoid the per-standard-deviation convention because the underlying titre distribution is not reported. One further caveat follows from the source's analytic design. The Tohoku Medical Megabank cohorts, which contain related individuals, were analysed with a linear mixed model, and the cohort-level results were then combined by inverse-variance weighted meta-analysis. The source does not state how the mixed-model coefficients were placed on the same scale as those from the remaining cohorts, so we treat the pooled coefficients as approximately on the log odds scale of the dichotomised phenotype and do not attempt any further rescaling.

Outcome effects are log odds ratios, which we present as odds ratios with 95% confidence intervals throughout. Where we compare exposures measured on different scales, as in the analyses of other pathogens, we compare direction and strength of evidence rather than magnitude, and say so at each point.

What the MR-Egger intercept can and cannot show here

The MR-Egger intercept is widely read as a test for pleiotropy, and a non-significant intercept as reassurance that none is present. That reading does not hold in this setting. The intercept detects bias only when the unwanted effects point predominantly in one direction and are unrelated to how strongly each proxy predicts the exposure, the second condition being the

InSIDE assumption. Proxies that all tag one haplotype violate it, because the same haplotype determines both how strongly a proxy predicts antibody response and how strongly it predicts disease. A non-significant intercept is therefore not evidence that no bias is present, and we do not treat it as such anywhere in this paper. The same reasoning applies to MR-PRESSO, which identifies variants that are outliers relative to the others and cannot identify a bias shared by all of them.

This is the reason we report estimators that tolerate correlated pleiotropy alongside the standard ones, and the reason the argument in the main text rests on partitioning the region, on colocalisation and conditional analysis, and on the control outcomes rather than on any pleiotropy diagnostic.

Selecting proxies in European data

It would be preferable to select proxies in the same population as the outcome. This is not viable here: the seven publicly available European antibody traits for *H. pylori* yield only two genome-wide significant variants between them, too few for an instrument. We therefore held the selection fixed at the 22 variants reported by the Japanese discovery study and looked each up in the European data regardless of its significance there. Selecting on significance in the European data and then testing in it would have introduced both a threshold artefact and the bias of selecting and testing in one sample.

Participant overlap between exposure and outcome

Overlap between the samples that supply the exposure and the outcome biases two-sample estimates towards the observational association, and the bias is larger when instruments are weak. The Japanese exposure and European outcome cohorts are ancestrally distinct. For Japanese outcomes, participant overlap requires comparison of the contributing cohorts and cannot be excluded solely because the exposure and outcome GWAS were published separately.

Overlap is also possible in the analyses that substitute other antibody traits for H. pylori, because those traits were measured in UK Biobank and UK Biobank contributes to some of the outcome meta-analyses. We report those analyses as a comparison of directions rather than of magnitudes for this reason among others, and we take advantage of the fact that the Alzheimer disease consortium provides both a version including biobank cohorts and a version excluding them, which allows the influence of such participants to be examined directly.

Multiple testing

For the primary H. pylori analyses, Benjamini-Hochberg correction was applied across the six outcomes within each of the five genomic proxy sets. The sets were not pooled into one testing family. The additional pathogen-antibody traits formed a separate family. Within each proxy set, Benjamini-Hochberg correction was applied across every antibody-trait and outcome combination (11 traits, 3 outcomes) for which an estimate could be obtained: 29 tests for the all-variant set, 21 for MHC excluded, 16 for MHC only, 12 for class I only and 8 for class II only. Combinations with no usable proxy at the selection threshold were excluded from the family rather than counted as tests, which is why the family sizes are smaller than 33. The H. pylori estimates that appear alongside these traits in Figure 4 keep the q values of the primary family and were not re-entered into the antibody-trait family. The peptic ulcer analyses and the between-outcome difference tests are reported with nominal P values. Nominal P values are distinguished from q values where the latter were calculated; not every supplementary comparison has a q value.

The five sets were applied to every exposure and every outcome, including the 18 additional pathogen antibody traits, before any result was inspected, so no set was chosen after seeing an estimate.

Alternative definitions of the region

Published definitions of the extended major histocompatibility complex differ. Our primary definition is chromosome 6, 25 to 34 megabases, with the class I sub-region taken as 29.6 to 31.5 megabases and the class II sub-region as 32.3 to 33.1 megabases, all on GRCh37. We repeated the analysis using an alternative published definition and report the result in Supplementary Table S2. The effect of the selection threshold on the proportion of proxies falling inside the region is given in Supplementary Table S5.

Attributing the signal

Classical HLA class II allele attribution

Individual-level data were not available, so classical alleles could not be imputed. We instead used the tag variants that the literature reports for each allele, and computed their correlation with each proxy in the European and East Asian reference panels, together with their association with each outcome in the datasets used elsewhere in this paper. Genomic positions of the tag variants were retrieved from Ensembl on GRCh37.

Two limitations follow and both are stated in the main text. What is measured is the correlation with a tag variant, not with the allele itself; and the fidelity with which a tag represents its allele is ancestry dependent, which is the same property the paper is about. The allele assignments in

Supplementary Table S15 are literature reports; the correlations and disease associations in that table are computed here. Readers should not treat the two as equally supported.

Approximate conditional analysis

Conditional effects follow the summary-statistic formulation of Yang and colleagues. Writing $v = 2f(1 - f)$ for a variant of effect-allele frequency f, and r for the correlation between the two variants, the effect of variant 1 conditional on variant 2 is

$$b1|2 = [b1 - r \sqrt{(v2 / v1)}\, b2] / (1 - r2), \qquad se1|2 = se1 / \sqrt{(1 - r2)}.$$

Because the conditioning is performed within a single genome-wide association study, the sample size enters both terms identically and cancels, which removes one source of error. Correlations were taken with alleles attached and aligned explicitly to the effect alleles of each dataset; where alleles could not be matched the pair was dropped rather than assigned by assumption. Pairs correlated above an absolute r of 0.975 were not reported because the conditional standard error becomes unstable. The 1/(1 − r2) factor was omitted from the conditional coefficients in an earlier version of this analysis, although the standard errors already included it; the coefficients and p values in Supplementary Table S16 have been recomputed by applying the omitted factor, and the standard errors are unchanged. The correction moves the reported conditional estimates further from the null without changing which of these two proxy associations reaches nominal significance: the two proxy associations that were abolished after conditioning remain so ($p = 0.27$ and $p = 0.44$), and the lead variants that retained their associations still do.

The method is approximate for logistic regression coefficients. The approximation is best when per-allele effects are small, which holds for the proxy and disease coefficients conditioned on here, but not for the multiple sclerosis lead variant, whose log odds ratio is large; the multiple sclerosis conditional estimates should therefore be read as indicative of direction rather than as accurate magnitudes. It also assumes that reference-panel linkage disequilibrium matches that of the study population, an assumption that is weaker in the major histocompatibility complex than elsewhere. We cannot determine the direction in which any residual mismatch would move these estimates, and we do not claim that it favours our interpretation.

How much a single class II signal accounts for

If a single causal variant in the class II sub-region acted on disease, and the proxies affected

disease only through their correlation with it, the disease effect of proxy *i* would be

$b_{GY,i} = \gamma\, r_i \sqrt{(v_{lead} / v_i)}$,

where γ is the effect of that variant, r_i its correlation with proxy *i*, and *v* is defined as above. Every quantity on the right except γ is taken from data. We estimated γ by weighted least squares through the origin, weighting by the inverse variance of the observed disease effect, and report the weighted R^2 as the share of the proxies' disease associations explained and a residual Q statistic as a test of whether one signal suffices.

Substituting the East Asian correlation for the European one, with γ and everything else held fixed, gives the estimate that the same causal structure would produce if the proxies were applied in the population where they were discovered. The ratio between the two describes sensitivity to replacing the LD correlations under this model; it is not a direct estimate of transport bias in an observed population.

Winner's curse

The proxies were selected because they exceeded a significance threshold in the discovery study, so their reported effects are inflated. We corrected each by conditional maximum likelihood, maximising the density of the observed effect conditional on selection at $p < 5 \times 10^{-8}$, and recomputed the instrumental-variable estimates with the corrected exposure effects.

The direction of this bias matters for interpretation. Because the ratio estimate divides the disease effect by the exposure effect, an inflated exposure effect shrinks each variant's ratio towards the null, and correcting the exposure effect moves that ratio away from it. The combined estimate does not have to follow, because the correction also enlarges the standard error of each corrected ratio and so changes the weights, most for variants discovered close to the threshold. In the European datasets the class II estimates moved further from the null after correction (Alzheimer disease 0.73 to 0.67; Parkinson disease 0.73 to 0.67), so winner's curse attenuates rather than generates those associations. The East Asian Parkinson disease estimates moved towards the null (class II 0.15 to 0.28) and lost nominal significance. Variants discovered close to the threshold shrink severely and their corrected ratios are unstable; the class I sets contain such variants. We report the correction as a sensitivity analysis and not as a primary result (Supplementary Table S18).

Software and reference data

Analyses used R 4.6.1 with TwoSampleMR 0.7.9, MendelianRandomization 0.10.0, coloc 5.2.3, susieR 0.14.2, MR-PRESSO 1.0, ieugwasr 1.1.0 and data.table 1.18.4. Linkage disequilibrium was taken from the 1000 Genomes Phase 3 European and East Asian panels through the OpenGWAS interface. Full version details are in Supplementary Table S11.

Supplementary Figures

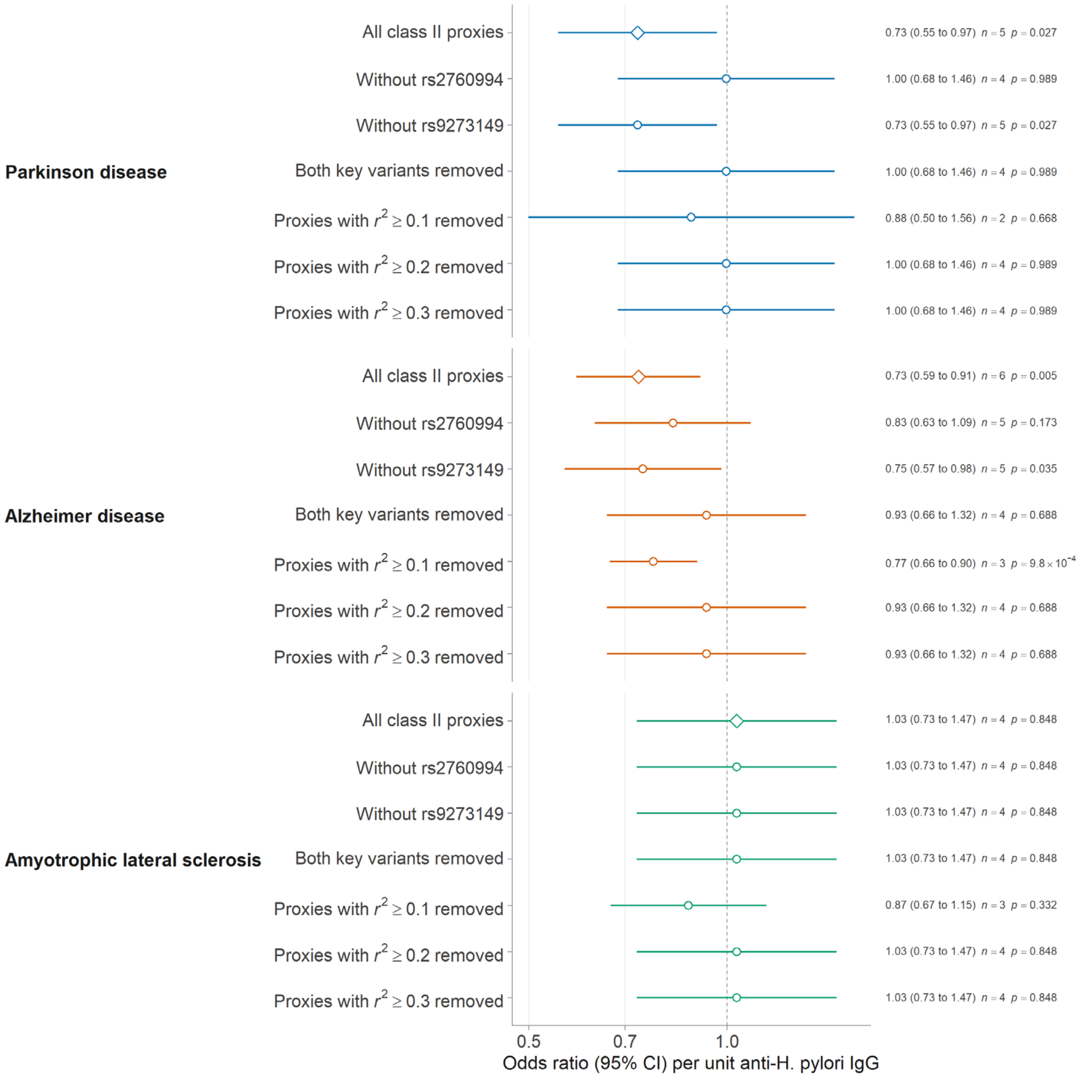

Supplementary Figure S1. Effect of removing the two variants that account for most of the association. Analyses were restricted to human leukocyte antigen class II variants. Diamonds show the primary estimate for each disease, and circles show estimates after variant removal. The final three rows within each disease block apply prespecified squared-correlation thresholds of 0.10, 0.20 and 0.30 to remove proxies in linkage disequilibrium with a leading disease variant. Values shown at the right are the odds ratio, 95% confidence interval, number of contributing variants and P value. In the Parkinson disease data set rs9273149 is not present without proxy substitution, so the row without that variant repeats the primary estimate and the row without both variants reflects the removal of rs2760994 alone.

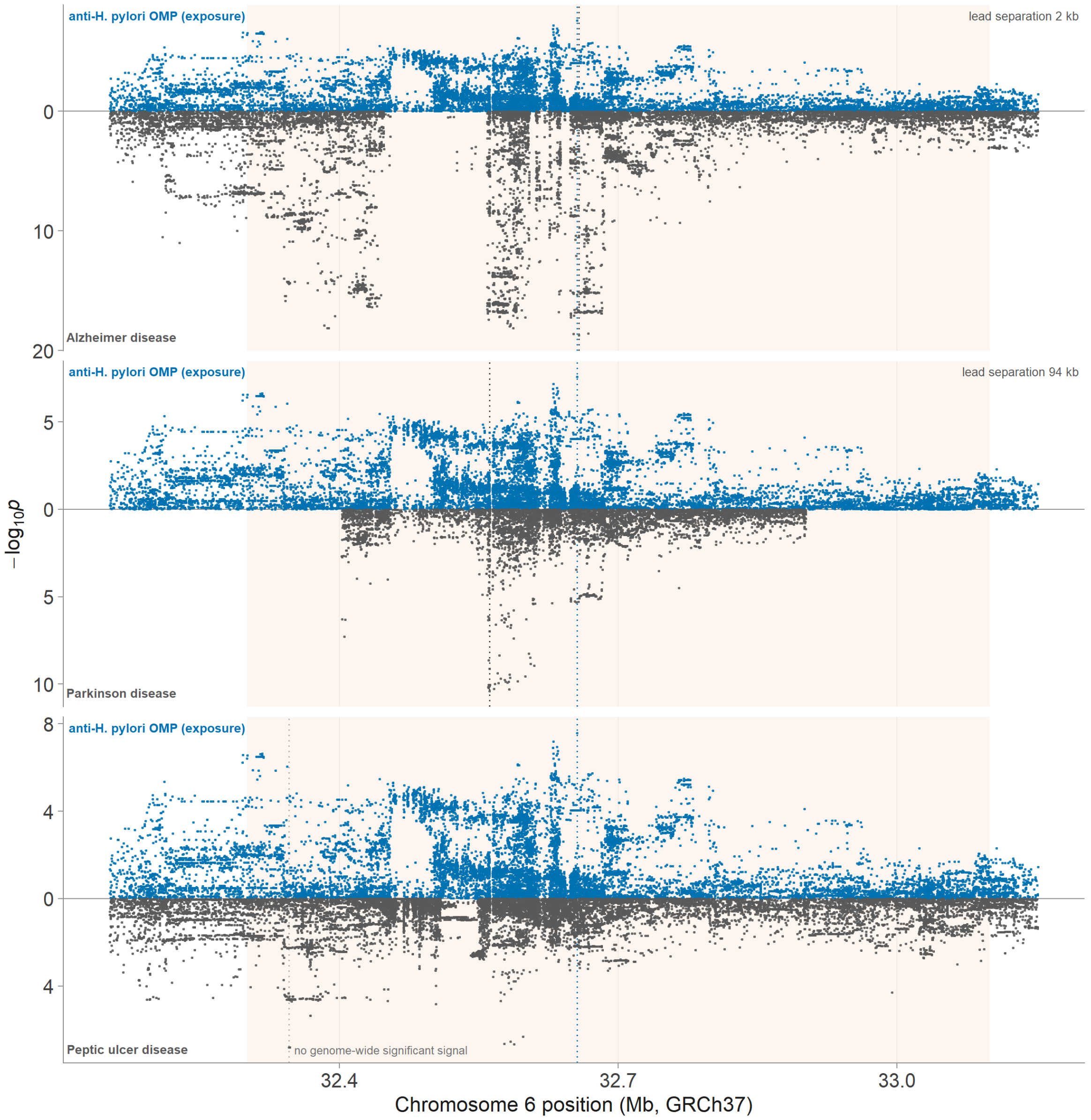

Dotted lines mark the leading variant of each trait. Shading marks HLA class II.

Supplementary Figure S2. Regional association patterns for the antibody response and disease outcomes. The anti-*H. pylori* outer membrane protein antibody association is plotted in the upper half of each plot and the corresponding disease association in the lower half, using a shared chromosomal axis. Dotted lines identify the leading variant for each trait, and shading identifies the human leukocyte antigen class II region. The leading Alzheimer disease and antibody-response variants are separated by 2 kilobases; formal colocalisation analysis nevertheless supports distinct causal variants.

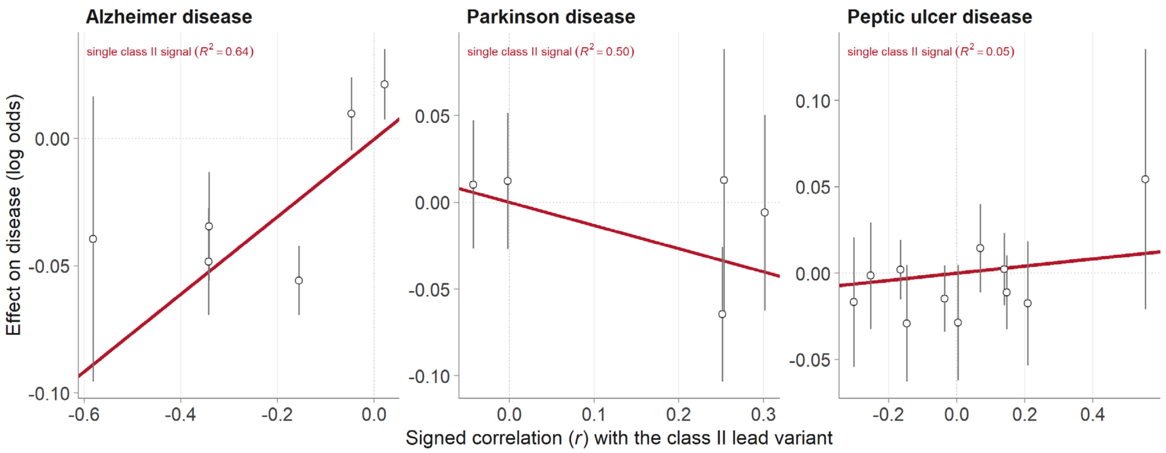

Supplementary Figure S3. Fit of a one-parameter human leukocyte antigen class II model. For each disease, the effect estimate for every class II proxy is plotted against the scaled predictor r × sqrt(v_lead/v_i), where r is the signed European LD correlation and v is the allele-dosage variance, together with the fitted one-parameter model. Points would lie on the fitted line if the leading variant fully accounted for the proxy-disease associations. Vertical bars show 95% confidence intervals. The weighted coefficient of determination is reported for each disease and in Supplementary Table S17. These are exploratory input sets: the Parkinson disease and peptic ulcer disease variants differ from the current primary sets and require reconciliation before primary-analysis interpretation.

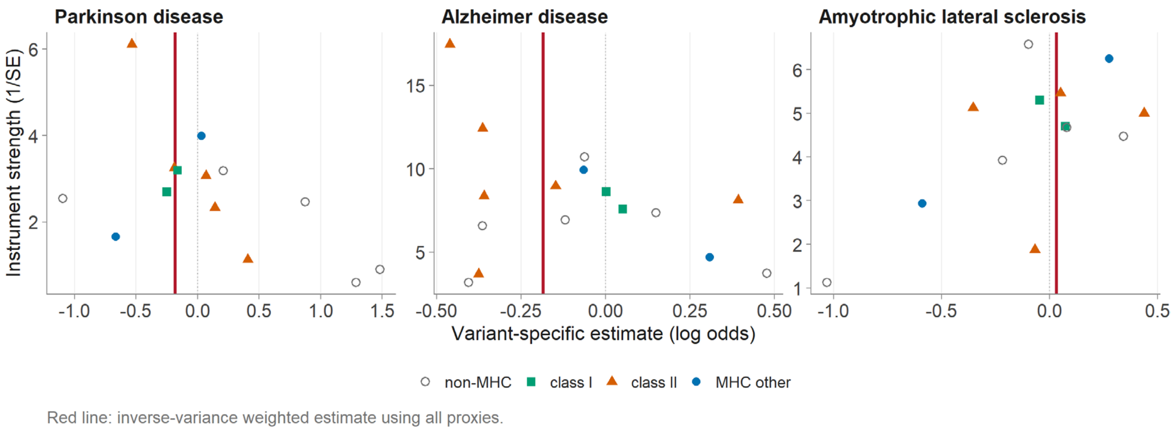

Supplementary Figure S4. Variant-specific estimates according to inverse standard error. The Alzheimer disease estimates are asymmetric, consistent with the MR-Egger intercept reported in Supplementary Table S2. Negative estimates arise predominantly from human leukocyte antigen class II variants, whereas variants outside the major histocompatibility complex are centred near the null. The updated Parkinson disease and amyotrophic lateral sclerosis estimates are also shown; visual asymmetry alone is not a formal test of pleiotropy. The red line shows the inverse-variance weighted estimate based on all variants.
